# Retinal Glial Cells in Von Hippel–Lindau Disease: A Novel Approach in the Pathophysiology of Retinal Hemangioblastoma

**DOI:** 10.3390/cancers14010170

**Published:** 2021-12-30

**Authors:** Elisabetta Pilotto, Giulia Midena, Tommaso Torresin, Gilda De Mojà, Maria Laura Bacelle, Alfonso Massimiliano Ferrara, Stefania Zovato, Edoardo Midena

**Affiliations:** 1 Department of Ophthalmology, University of Padova, 35128 Padova, Italy; tommaso.torresin@unipd.it (T.T.); marialaura.bacelle@gmail.com (M.L.B.); edoardo.midena@unipd.it (E.M.); 2 ERN-EYE Center, University Hospital of Padova, 35128 Padova, Italy; 3 IRCCS-Fondazione Bietti, 00198 Rome, Italy; giulia.midena@fondazionebietti.it; 4 Oftalmico Hospital, ASST Fatebenefratelli Sacco, 20121 Milano, Italy; gildademo@gmail.com; 5 Familial Tumor Unit, Veneto Institute of Oncology IOV-IRCCS, 35128 Padova, Italy; massimiliano.ferrara@iov.veneto.it (A.M.F.); stefania.zovato@iov.veneto.it (S.Z.)

**Keywords:** Von Hippel–Lindau disease, retinal hemangioblastoma, OCT, retinal macroglia, astrocytes, retinal microglia

## Abstract

**Simple Summary:**

The in vivo optical coherence tomography analysis of the biomarkers of retinal microglia and macroglia in Von Hippel–Lindau disease represents an innovative field of research. The different behavior of these glial cells in Von Hippel–Lindau patients provides new data regarding the pathophysiology of retinal hemangioblastoma, the most common ocular manifestation of this hereditary disorder. Moreover, these biomarkers show a different behavior in Von Hippel–Lindau patients in relation to the presence or absence of retinal hemangioblastoma. Therefore, we can hypothesize that retinal hemangioblastoma is mainly due to the activation of macroglia by previously activated microglial cells.

**Abstract:**

Background: Von Hippel–Lindau (VHL) disease is a neoplastic syndrome caused by a mutation of the VHL tumor suppressor gene. Retinal hemangioblastoma (RH) is a vascularized tumor and represents the most common ocular manifestation of this disease. At the retinal level, VHL protein is able to regulate tumor growth, angiogenic factors, and neuroinflammation, probably stimulating retinal glial cells. The aim of the present study was to analyze in vivo the optical coherence tomography (OCT) biomarkers of retinal macroglia and microglia in a cohort of VHL patients. Methods: The mean thicknesses of macular retinal nerve fiber layer (mRNFL), ganglion cell layer (GCL), and peripapillary retinal nerve fiber layer (pRNFL) were measured with OCT as biomarkers of retinal macroglia. OCT images were also analyzed to detect and quantify hyperreflective retinal foci (HRF), a biomarker of retinal activated microglia. Results: 61 eyes of 61 VHL patients (22 eyes (36.07%) with peripheral RH and 39 eyes (63.93%) without RH) and 28 eyes of 28 controls were evaluated. pRNFL was thinner in VHL patients (*p* < 0.05) and in VHL without RH (*p* < 0.01) compared to controls, and thicker in VHL patients with RH than in those without RH (*p* < 0.05). The thickness of mRNFL (*p* < 0.0001) and GCL (*p* < 0.05) was reduced in VHL patients and in VHL without RH compared to controls, whereas mRNFL (*p* < 0.0001) and GCL (*p* < 0.05) were increased in VHL patients with RH compared to those without RH. HRF were significantly higher in number in VHL patients and in VHL without RH, than in controls, and significantly lower (*p* < 0.05) in the eyes of VHL patients with RH, than in those without RH. Conclusions: The OCT analysis, which detects and allows to quantify the biomarkers of retinal microglia (HRF) and macroglia (pRNFL, mRNFL and GCL), showed a different behavior of these two retinal glial cells populations in VHL patients, related to the presence or absence of peripheral RH. These data allow to hypothesize a novel pathophysiologic pathway of retinal hemangioblastoma in VHL disease.

## 1. Introduction

Von Hippel–Lindau (VHL) disease is a neoplastic syndrome caused by a mutation of the VHL tumor suppressor gene [1,2,3]. VHL gene encodes a tumor suppressor protein (pVHL) which forms a protein complex responsible for the degradation of hypoxia-inducible factors (HIF). When pVHL is absent, HIF expression increases with subsequent over-production of several cytokines and growth factors, mainly VEGF [4]. In addition to this mechanism, cellular adaptation to the hypoxic response requires the integration with the NOTCH signaling pathway. NOTCH signaling leads to the transcriptional activation of target genes, such as Hes-1 and Hes-5, which play a crucial role in neurogenesis, gliogenesis, neuritogenesis, and in the development of sensory organs [5,6]. All these proliferative, angiogenic and glial regulative mechanisms lead to the development of various tumors including hemangioblastomas, typical vascularized hamartomatous lesions localized in the central nervous system and in the retina in VHL patients [4,5,7,8,9,10]. Retinal hemangioblastoma (RH) is a lesion that develops in the peripheral or in the juxtapapillary retina. It is histopathologically similar to central nervous system hemangioblastoma and composed by different cell types: endothelial cells and pericytes, which form a network of vascular channels, and stromal cells, which are believed to be the neoplastic component of the tumor [3,10,11,12,13,14,15]. The histogenesis of these RH stromal cells is still an enigma and several theories have been suggested, mainly hypothesizing specific glial cells, the astrocytes [10,11,12,13].

The application of clinical optical coherence tomography (OCT) allows to analyze the retina, layer by layer, identifying alterations in specific cell populations, and to identify the presence of hyperreflective retinal foci (HRF), considered a clinical biomarker of activated retinal microglial cells [15,16,17,18,19,20,21,22]. Recent OCT studies on VHL patients have shown a generalized thinning of the peripapillary retinal nerve fiber layer (pRNFL), except when the eye harbors RH [15]. In these last cases pRNFL increases in thickness. These findings have raised the hypothesis that the thickening of pRNFL is the consequence of an activation and proliferation of cellular elements normally resident in this layer, such as retinal astrocytes [23,24,25,26,27,28,29].

The aim of this study was to comprehensively analyze and correlate in vivo the OCT biomarkers of retinal macroglia, through the structural characteristics of peripapillary retinal nerve fiber layer (pRNFL), macular retinal nerve fiber layer (mRNFL) and of ganglion cells layer (GCL), and retinal microglia, through the quantification of HRF in VHL eyes, with or without peripheral RH.

## 2. Materials and Methods

### 2.1. Participants

This was a cross-sectional study. VHL patients, admitted to the Familial Tumor Unit of the Veneto Institute of Oncology (IOV-IRCCS), were consecutively included. The ophthalmological evaluation included: best corrected visual acuity (BCVA), with standard Early Treatment Diabetic Retinopathy Study (ETDRS) charts; slit-lamp examination of anterior and posterior (90-diopter-lens) segment; intraocular pression measurement; indirect ophthalmoscopy. To avoid factors confounding detailed OCT analysis of macroglia and microglia biomarkers, by tissue changes, we excluded: RH located in the posterior pole or in the juxtapapillary area; others retinal disorders (such as diabetic and hypertensive retinopathy and/or maculopathy, uveitis, and glaucoma); retinal pigment epithelium alterations or laser scars located in the posterior pole; epiretinal macular membrane; anterior segment disorders; refractive errors ≥6 diopters. A control group, composed by age- and gender-matched patients underwent the same examinations.

### 2.2. OCT

OCT was performed with Spectralis HRA + OCT (Heidelberg Engineering, Heidelberg, Germany). After pupil dilatation, obtained with 1% tropicamide solution, in a dark room, late in the morning, with the in-built eye-tracker activated, the following SD-OCT scans were obtained: a peripapillary 3.5 mm ring N-RNFL (N-site/axonal menu) scan, centered onto the optic nerve head, to automatically provide the pRNFL thickness value. pRNFL thickness was defined both as mean pRNFL (pRNFL) and as sectorial pRNFL (Temporal, T-pRNFL; Temporal Superior, TS-pRNFL; Nasal Superior, NS-pRNFL; Nasal, N-pRNFL; Nasal Inferior, NI-pRNFL; Temporal Inferior, TI-pRNFL; Papillo Macular Bundle, PMB) [15,30]. Moreover, a single horizontal scan (180° line scan, 9 mm length, automated real time (ART) set at 100 frames) and a macular map of 20° × 20° (5.8 × 5.8 mm; 97 B-scans separated by 60 micron) were obtained. Spectralis is able, through the automatic retinal layering, to detect seven retinal layers: mRNFL, GCL, inner plexiform layer (IPL), inner nuclear layer (INL), outer plexiform layer (OPL), Henle fiber layer with outer nuclear layer (ONL), and outer retinal layers (between external limiting membrane and retinal pigment epithelium–Bruch membrane complex). In case of uncertain interpretation of the boundaries, manual refinement of the layers was performed. For each of the nine ETDRS areas (central circle 1 mm in diameter, and two external rings 3 and 6 mm in diameter), the mean retinal thickness was calculated. Mean retinal thickness of mRNFL and GCL were measured. A skilled technician performed all scans and checked each image after acquisition to detect any motion artifacts or segmentation errors, in these cases acquisition was repeated. After segmentation of retinal layers, red vertical lines were traced at 1500 μm nasally and temporally to the center of the fovea, allowing to analyze 3000 μm of the central single 180° line OCT scan of the map for HRF detection and quantification. To exclude the presence of retinal artifacts, vessels, and other pathologic conditions interfering with HRF detection the en face complete reconstruction was applied. HRF were evaluated on the central B-scan passing through the fovea, which was displayed on a monitor simultaneously with and below an en face map. HRF were defined as discrete intraretinal hyperreflective foci ≤30 μm in size, with reflectivity similar to nerve fiber layer and without back shadowing [18]. HRF were identified and counted as follows: in the full thickness retina—from the boundary between RNFL/GCL to the external limiting membrane (ELM); in the Inner Retina (IR)—all layers between the boundary RNFL/GCL and the lower limit of the outer plexiform layer (OPL); and in the Outer Retina (OR)—between the upper limit of the outer nuclear layer (ONL) and the ELM.

### 2.3. Statistical Analysis

All measures were described according to the usual methods of descriptive statistics: the qualitative parameters were expressed in terms of absolute and relative frequency (percentage), the quantitative ones in terms of arithmetic mean, standard deviation and interval of variation (minimum, maximum). The Gaussian distribution of the parameters in the sample groups was verified by the Shapiro–Wilk test. Gender and age were compared with Fisher’s exact test and *t*-Student test for independent samples, respectively. Concerning macular thickness, since the measurement was replicated in the nine retinal quadrants, the model used was that for repeated measurements with two main factors (Group and Quadrant) with interaction (Group*Quadrant) in order to compare the profiles Retinal (Quadrants) of Groups: Tukey–Kramer adjustment for multiple comparisons was used for this analysis. The inferential analysis concerned HRF of the IR, OR and full retina. The mean values of the measured parameters were compared between VHL patients and controls, VHL patients without RH and controls, VHL patients with RH and controls and VHL patients with and without RH, using ANOVA model (Group). Finally, the correlations between BCVA and CRT, and between pRNFL and number of HRF in IR were evaluated, by means of separate analyzes for total VHL patients, VHL patients without RH, VHL patients with RH and controls. For this purpose, a generalized linear regression model adjusted for the subject’s age was used, with tests on the statistical significance of the regression coefficient. All analyses were performed using SAS^©^ 9.4 software (SAS Institute, Cary, NC, USA). Statistical tests were interpreted as significant if *p* < 0.05.

### 2.4. Ethics Statement

Informed consent was obtained from each subject; data collection was compliant with the tenets of the Declaration of Helsinki. The approval from the Ethics Committee of our Institution was obtained (Prot. 59n/AO/20).

## 3. Results

### 3.1. Population Characteristics

In this study, 61 eyes of 61 VHL patients and 28 eyes of 28 healthy subjects were included. In the VHL group, composed by 32 females (52.46%) and 29 males (47.54%), mean age was 39.8 ± 14.3 years; in the control group, including 17 females (60.71%) and 11 males (39.29%), mean age was 40.0 ± 11.8 years (*p* = 0.9579). Among females, the mean age was 42.8 ± 10.0 years in the VHL group and 43.3 ± 9.3 years in the control group. Among males, the mean age was 36.3 ± 11.5 years in VHL group and 36.2 ± 10.5 years in the control group. There was no significant difference in gender (*p* = 0.4672) between the study groups. The VHL group included 56 right eyes and 5 left eyes. Twenty-two eyes (36.07%) were affected by peripheral RH, while 39 eyes (63.93%) were free of retinal lesions. Based on genetic classification, 48 patients (78.69%) had type 1 VHL mutations, 13 (21.31%) had type 2 VHL mutations: one patient (1.64%) with type 2A VHL, nine (14.75%) with type 2B VHL, and three (4.92) type 2C VHL. Comparative analysis based on these genetic subtypes was not performed due to the limited sample size of patients with type 2 VHL. Genetic mutations are listed in Table 1.

### 3.2. Peripapillary Retinal Nerve Fiber Layer Thickness

pRNFLvalues in VHL patients and controls are summarized in Table 2. pRNFL was statistically thinner in the temporal-superior sector in VHL patients than in controls (*p* = 0.0227) and in VHL patients without RH compared to controls (*p* = 0.0029). pRNFL was thicker in VHL patients with RH compared to those without RH, especially in the temporal-superior sector (*p* = 0.0181), in the temporal sector (*p* = 0.0587) and in temporal-inferior sector (*p* = 0.0647), as reported in Table 3.

### 3.3. Macular Layers Thickness and Volume Analysis

#### 3.3.1. Retinal Nerve Fiber Layer Analysis

mRNFL volume was reduced in VHL patients (*p* = 0.0554) and in VHL patients without RH (*p* = 0.0270) compared to controls, as reported in Table 4. The thickness of mRNFL layer was reduced in the superior-outer sector in VHL (*p* < 0.0001) and in VHL subjects without RH (*p* < 0.0001) and in the nasal-outer sector in VHL (*p* = 0.0301) and in VHL subjects without RH (*p* = 0.0007). mRNFL thickness in the superior-inner sector was reduced the VHL group (*p* = 0.0585) and in VHL patients without RH (*p* = 0.0335), compared to controls. In VHL patients with RH only the superior-outer sector of mRNFL was significantly thinner (*p* = 0.0111) than in controls. Comparing VHL patients with RH to those without RH, mRNFL was thicker in the nasal-outer sector in patients with retinal lesion (*p* < 0.0001). Statistical significance details are reported in Table 5.

#### 3.3.2. Ganglion Cells Layer Analysis

GCL volume and thickness showed a different behavior in VHL subjects, in controls and in VHL according to the presence or absence of RH. In particular, as reported in Table 6, GCL volume was reduced in VHL subjects without RH versus controls (*p* = 0.0412). Moreover, GCL thickness was reduced in the temporal-outer sector in VHL (*p* = 0.0212) and in VHL patients without RH (*p* = 0.0073) compared to controls. Furthermore, thickness reduction was assessed in the temporal-inner sector in VHL without RH (*p* = 0.0381) versus controls. Comparing VHL patients with and without RH there was a increase in the thickness in the nasal-outer sector (*p* = 0.0580) and in the volume in VHL subjects with RH (*p* = 0.0561). Statistical significance details are reported in Table 7.

### 3.4. Best Corrected Visual Acuity and Central Retinal Thickness

BCVA was statistically significant lower in VHL subjects compared to controls (*p* = 0.0007). Comparing VHL patients with and without RH to controls BCVA was lower (*p* = 0.0100 and *p* = 0.0461, respectively). Comparing VHL patients without RH to those with RH no differences in BCVA were found. The CRT was 273.0 ± 18.2 μm in the VHL group and 272.6 ± 18.4 μm in the controls, without a statistically significant difference between the study groups. Statistical significance details are reported in Table 8 and Table 9. Moreover, no correlation was identified between BCVA and CRT.

### 3.5. Quantification and Distribution of HRF

**Figure 1:**
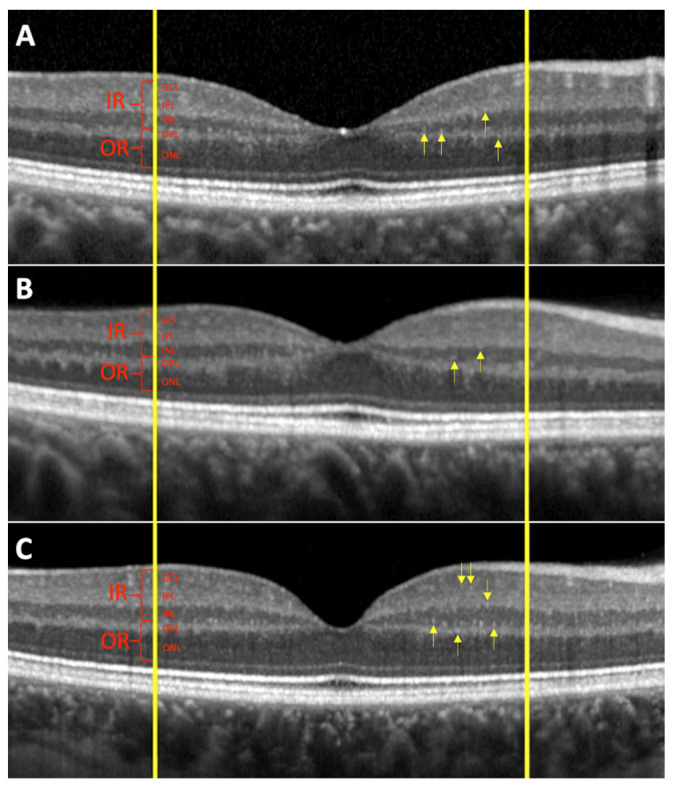
Hyperreflective retinal foci (arrows) at the OCT linear scan in one healthy control (**A**), in one VHL eye with retinal hemangioblastoma (**B**), and in one VHL eye without retinal hemangioblastoma (**C**) are shown. IR: Inner Retina; OR: Outer Retina; GCL: Ganglion Cell Layer; IPL: Inner Plexiform Layer; INL: Inner Nuclear Layer; OPL: Outer Plexiform Layer; ONL: Outer Nuclear Layer.

The mean HRF number in the total retina was 36.0 ± 9.3 in VHL eyes and 32.9 ± 8.9 in controls, as reported in Table 10. The difference, although showing higher values in VHL patients compared to controls, was not statistically significant (*p* = 0.1505). The mean number of HRF in the IR was statistically higher in the VHL patients compared to controls (*p* = 0.0554). On the other hand, the number of HRF in the OR did not differ between the VHL group and the controls, although the values were lower in VHL patients. In the VHL group without RH, there was a statistically significant increase in HRF in the full retina (*p* = 0.0302) and in the IR (*p* = 0.0121) compared to controls. Lower values of HRF in the IR and OR in VHL patients with RH were found, compared to the group of VHL without RH, while *p*-value was at the limit of significance between the two groups concerning the number of HRF in the total retina (*p* = 0.0677). Statistical significance details of HRF are reported in Table 11. In Figure 1 are shown HRF in VHL with and without RH and in controls.

**Figure 2:**
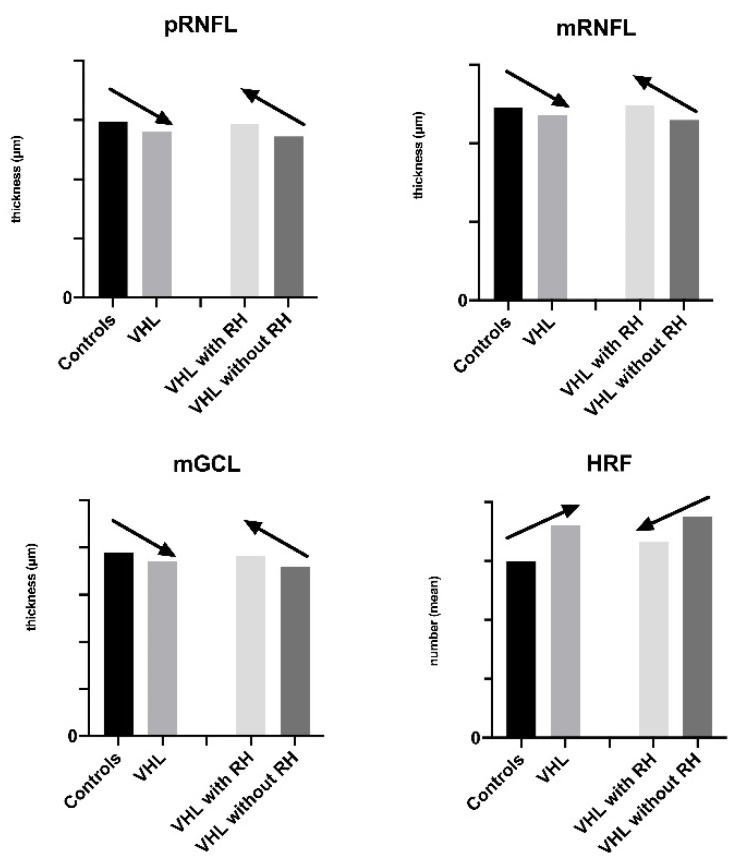
Trend of retinal thickness and of hyperreflective retinal foci expression in the analyzed groups. VHL: Von Hippel–Lindau; RH: Retinal Hemangioblastoma; pRNFL: peripapillary Retinal Nerve Fiber Layer; mRNFL: macular Retinal Nerve Fiber Layer; GCL: Ganglion Cell Layer; HRF: Hyperreflective Retinal Foci.

The above-mentioned OCT differences (pRNFL, mRNFL, GCL, and HRF) between controls and VHL groups and between VHL patients with and without RH are reported in Figure 2.

## 4. Discussion

From its discovery, VHL disease tumor suppressor gene has proved to be of a wide scientific interest. Maxwell et al. demonstrated the fundamental role of pVHL in the regulation of HIF, and over the years the details of the pVHL–HIF pathway (implicating VEGF overexpression) were elucidated [31]. Moreover, pVHL has HIF-independent functions, including assembly and regulation of the extracellular matrix, microtubule stabilization, regulation of apoptosis, control of cell senescence and transcriptional regulation [1,2,3,4,5,6]. Despite the progress in VHL genetic and biology studies, the pathophysiology of RH is still unclear. RH is the most characteristic lesion of VHL eye disease and, in spite of its benign nature, RH remains a major cause of visual impairment in these patients. Grossniklaus et al. focused on the histopathologic, immunohistochemical, and ultrastructural features of RH and indicated that RH consists of endothelial-lined vascular channels in a stroma composed of intrinsic polyhedral and foamy glial cells, and also reactive spindle-shaped glial cells [10]. It is actually well known that human retina contains three types of glial cells: microglia and two types of macroglia, namely, astrocytes and Müller cells [32]. Nowadays, with the development of retinal imaging, OCT analysis is able to assess and characterize the in vivo biomarkers of macroglia (quantitative evaluation of some retinal layers) and microglia (HRF) [15,16,17,18,19,20,21,22]. Therefore, we analyzed the specific biomarkers of these cell populations in VHL subjects, in particular, focusing on the presence/absence of peripheral RH. Our results showed an overall thinning of pRNFL, mRNFL, and GCL retinal layers in VHL patients compared to controls, probably due to the macular and peripapillary perfusion impairment previously demonstrated [15,33]. We therefore analyzed how pRNFL, mRNFL, and GCL behaved, comparing VHL patients according to the presence or absence of RH. We demonstrated that pRNFL thickness was increased in VHL patients with RH compared to patients without RH. Furthermore, in patients with RH there was an increase in the thickness of mRNFL and GCL and in the volume of the latter. These data confirm our previous study, which showed that pRNFL was significantly thicker in the VHL eyes with RH compared to those without it, especially in the temporal sector, with no variations in retinal perfusion [15]. Therefore, we hypothesize that this increase in thickness is due by an activation and proliferation of other cellular elements located in this retinal layer. According to the literature, RNFL is composed of nerve fibers, vessels, and cells (mainly astrocytes and Müller cells, from 18% to 42%) and the thickness of this layer is proportional to density of the glial nuclei [34,35]. These results could contribute to validate the hypothesis that astrocytes are the most represented glial cells in the VHL population with RH and that an increase in pRNFL, mRNFL, and GCL, retinal layers mostly containing astrocytes, might be related to the formation of RH. However, a histopathologic confirmation should be necessary. Considering retinal microglia, the present study demonstrated an increase in HRF in VHL subjects compared to controls, in particular in the IR. This increase in number is a consequence of microglia activation, as previously reported in other retinal and systemic diseases. In particular, VEGF overexpression, due to the mutation of pVHL, might contribute to induce the activation of microglia in VHL patients [1,2,3,4,5,6,33,36,37,38,39,40]. We therefore hypothesize that pVHL alterations determine a “pre-activation” of the resident microglia. Moreover, the analysis of HRF based on the presence or absence of RH showed that HRF were lower in eyes with than in those without RH. These data seem to show an opposite behavior of macroglia (pRNFL, mRNFL, and GCL) and of microglia (HRF) in VHL patients compared to controls. In subjects with RH, there was a reduction in microglia (HRF) and an increase in macroglia biomarkers (increase of pRNFL, mRNFL, and GCL) compared to VHL subjects without RH. Therefore, it seems that the presence of RH affects microglia and macroglia in an opposite way. Recently, Liu et al., studying the interactions between microglia (M) and astrocytes (A) in the neurovascular unit, highlighted the presence of two phenotypes for each of these populations: one pro-inflammatory (M1 and A1) and one anti-inflammatory (M2 and A2). During neuroinflammation there is activation of the pro-inflammatory M1 population which, through the production of inflammatory signals (IL-1α and TNFα), stimulates the pro-inflammatory A1 population, thus amplifying neuroinflammation. Subsequently, the M1 phenotype switches to M2, through local intra and extracellular signals, with secondary production of anti-inflammatory signals, such as IL-10, whose receptor (IL-10R) is mainly expressed in A2 astrocytes, which are activated and proliferate. This connection (IL-10 with IL-10R) determines the production of TGFβ which reduces the activity of pro-inflammatory microglia, in order to moderate an exaggerated inflammatory response [41]. These data show that the expression of different phenotypes of microglia and astrocytes represents a dynamic change, secondary to the presence of pro- and anti-inflammatory signals. We can therefore hypothesize that, according to Knudson’s two-hit hypothesis of tumorigenesis already confirmed in VHL, the second hit in the retinal cell, as described by Chang in 1998, occurs in the proliferating glial population and, in particular, in astrocytes, which represent the tumoral component of the hemangioblastoma itself [42,43]. Moreover, it is interesting to underline that pRNFL thickness changes occurred mainly in the temporal sector, a classic location of peripapillary RH [44,45]. Therefore, its increase in thickness could be assumed as an imaging biomarker of RH development.

## 5. Conclusions

The biomarkers of macroglia and microglia showed a different behavior in VHL patients in relation to the presence or absence of peripheral RH. The thickening of the RNFL and GCL layers, due to macroglia activation and proliferation, is observed in VHL eyes with RH. According to literature, macroglia is mainly composed by astrocytes and in RH the tumor stromal cells are macroglial in origin. Instead, the increase in HRF, an expression of microglial activation, characterizes VHL eyes in the absence of RH. In conclusion, we can assume that RH is mainly due to the activation of astrocytes, harboring a second mutation (second hit), by previously activated microglial cells. Follow-up studies might evaluate the role of these OCT biomarkers in VHL retinal disease.

## Figures and Tables

**Table 1:** Mutation reported in the VHL group.

Mutation	Patients (Number, %)
c.485 G > T p.Cys162Phe	1, 1.6
c.202C > T p.Ser68Pro	1, 1.6
c.257C > T p.Pro86Leu	7, 11.5
c.277G > A p.Gly93Ser	2, 3.3
c.277G > C p.Gly93Arg	5, 8.2
c.314_315insC	2, 3.3
c.407T > C p.Phe136Ser	1, 1.6
c.456_457del AC p.Thr152fs	1, 1.6
c.462A > Cp.Pro154 = r.341_463del	1, 1.6
c.463 + 2T > C	1, 1.6
c.464-1G > A	2, 3.3
c.481C > T p.Arg161 *	2, 3.3
c.482G > A p. Arg161Gly	1, 1.6
c.486C > G p.Cys162Trp	4, 6.6
c.499C > T p.Arg167Trp	2, 3.3
c.548C > A p.Ser183 *	2, 3.3
c.551T > C p.Leu184Arg	1, 1.6
c.552 del A p. R113RfsX45	2, 3.3
c.563T > G p.Leu188Arg	1, 1.6
c.640T > G p. * 213Gly	1, 1.6
Complete deletion	7, 11.5
Exon 1 deletion	4, 6.6
Exon 2 deletion	1, 1.6
Exons 1 and 2 deletion	2, 3.3
Exon 3 deletion	4, 6.6
Exons 2 and 3 deletion	3, 5.0

* = stop codon.

**Table 2:** pRNFL thickness in the analyzed groups.

pRNFL	VHL	VHL with RH	VHL without RH	Controls
pRNFL (μm: mean ± SD)	101.1 ± 10.6	102.8 ± 8.2	100.1 ± 11.8	103.9 ± 7.0
T-pRNFL (μm: mean ± SD)	68.2 ± 10.1	71.4 ± 7.5	66.4 ± 10.9	69.9 ± 9.9
TS-pRNFL (μm: mean ± SD)	140.1 ± 17.4	146.9 ± 15.3	136.3 ± 17.6	148.9 ± 14.2
NS-pRNFL (μm: mean ± SD)	111.6 ± 25.5	113.2 ± 22.8	110.7 ± 27.2	108.0 ± 19.0
N-pRNFL (μm: mean ± SD)	78.5 ± 16.6	77.5 ± 11.6	79.0 ± 18.9	82.7 ± 13.3
NI-pRNFL (μm: mean ± SD)	118.7 ± 23.0	113.6 ± 15.5	121.5 ± 26.0	122.4 ± 18.8
TI-pRNFL (μm: mean ± SD)	145.3 ± 18.8	151.4 ± 17.2	141.8 ± 19.0	147.1 ± 16.8
PMB (μm: mean ± SD)	52.6 ± 8.1	55.0 ± 6.5	51.2 ± 8.6	52.8 ± 6.9

pRNFL: mean Peripapillary Retinal Nerve Fiber Layer; VHL: Von Hippel–Lindau; RH: Retinal Hemangioblastoma; T-pRNFL: Temporal sector of peripapillary Retinal Nerve Fiber Layer; TS-pRNFL: Temporal-Superior sector of peripapillary Retinal Nerve Fiber Layer; NS-pRNFL: Nasal-Superior sector of peripapillary Retinal Nerve Fiber Layer; N-pRNFL: Nasal sector of peripapillary Retinal Nerve Fiber Layer; NI-pRNFL: Nasal-Inferior sector of peripapillary Retinal Nerve Fiber Layer; TI-pRNFL: Temporal-Inferior sector of peripapillary Retinal Nerve Fiber Layer; PMB: Papillo Macular bundle.

**Table 3:** Statistical significance (*p*-value) of pRNFL thickness in the analyzed groups.

pRNFL	VHL vs. Controls	VHL without RH vs. Control	VHL with RH vs. Control	VHL without RH vs. VHL with RH
RNFL	0.2131	0.1086	0.6360	0.3422
T-pRNFL	0.4615	0.1835	0.6359	0.0587
TS-pRNFL	**0.0227**	**0.0029**	0.6355	**0.0181**
NS-pRNFL	0.5074	0.6494	0.3813	0.7177
N-pRNFL	0.2443	0.3833	0.1832	0.7065
NI-pRNFL	0.4595	0.8827	0.0929	0.1405
TI-pRNFL	0.6603	0.2434	0.3695	0.0647
PMB	0.8931	0.4223	0.3238	0.0793

pRNFL: mean Peripapillary Retinal Nerve Fiber Layer; VHL: Von Hippel–Lindau; RH: Retinal Hemangioblastoma; T-pRNFL: Temporal sector of peripapillary Retinal Nerve Fiber Layer; TS-pRNFL: Temporal-Superior sector of peripapillary Retinal Nerve Fiber Layer; NS-pRNFL: Nasal-Superior sector of peripapillary Retinal Nerve Fiber Layer; N-pRNFL: Nasal sector of peripapillary Retinal Nerve Fiber Layer; NI-pRNFL: Nasal-Inferior sector of peripapillary Retinal Nerve Fiber Layer; TI-pRNFL: Temporal-Inferior sector of peripapillary Retinal Nerve Fiber Layer; PMB: Papillo Macular Bundle. *p*-value: statistically significant values (*p* < 0.05) in bold.

**Table 4:** Volume and thickness of mRNFL in the analyzed groups.

mRNFL	VHL	VHL without RH	VHL with RH	Controls
mRNFL-Vol (mm^3^: mean ± SD)	0.889 ± 0.077	0.876 ± 0.075	0.912 ± 0.077	0.929 ± 0.114
mRNFL-C (μm: mean ± SD)	12.6 ± 2.0	13.0 ± 1.5	12.0 ± 2.6	12.4 ± 2.2
mRNFL-SI (μm: mean ± SD)	23.6 ± 2.7	23.2 ± 2.6	24.3 ± 2.9	25.2 ± 2.9
mRNFL-NI (μm: mean ± SD)	20.7 ± 1.8	20.5 ± 1.8	21.1 ± 1.6	20.5 ± 1.7
mRNFL-II (μm: mean ± SD)	25.0 ± 2.6	24.8 ± 2.7	25.2 ± 2.4	24.8 ± 2.4
mRNFL-TI (μm: mean ± SD)	16.7 ± 1.0	16.8 ± 1.1	16.7 ± 0.8	16.9 ± 1.2
mRNFL-SO (μm: mean ± SD)	35.8 ± 4.0	35.4 ± 3.7	36.6 ± 4.5	39.6 ± 5.8
mRNFL-NO (μm: mean ± SD)	47.2 ± 5.9	45.9 ± 5.8	49.6 ± 5.5	49.1 ± 8.2
mRNFL-IO (μm: mean ± SD)	39.2 ± 5.1	38.7 ± 5.0	40.0 ± 5.4	40.4 ± 6.6
mRNFL-TO (μm: mean ± SD)	18.1 ± 0.9	18.1 ± 1.0	18.2 ± 0.9	18.5 ± 1.3

RNFL: Retinal Nerve Fiber Layer; VHL: Von Hippel–Lindau; RH: Retinal Hemangioblastoma; SD: Standard Deviation; mRNFL-Vol: macular Volume of Retinal Nerve Fiber Layer; mRNFL-C: Central sector of macular Retinal Nerve Fiber Layer; mRNFL-SI: Superior-Inner sector of macular Retinal Nerve Fiber Layer; mRNFL-NI: Nasal-Inner sector of macular Retinal Nerve Fiber Layer; mRNFL-II: Inferior-Inner sector of macular Retinal Nerve Fiber Layer; mRNFL-TI: Temporal-Inner sector of macular Retinal Nerve Fiber Layer; mRNFL-SO: Superior-Outer sector of macular Retinal Nerve Fiber Layer; mRNFL-NO: Nasal-Outer sector of macular Retinal Nerve Fiber Layer; mRNFL-IO: Inferior-Outer sector of macular Retinal Nerve Fiber Layer; mRNFL-TO: Temporal-Outer sector of macular Retinal Nerve Fiber Layer.

**Table 5:** Statistical significance (*p*-value) of mRNFL thickness in the analyzed groups.

mRNFL	VHL vs. Controls	VHL without RH vs. Controls	VHL with RH vs. Controls	VHL without RH vs. VHL with RH
mRNFL-Vol	**0.0554**	**0.0270**	0.5964	0.0809
mRNFL-C	0.7662	0.5036	0.7206	0.2448
mRNFL-SI	0.0585	**0.0335**	0.4261	0.2150
mRNFL-NI	0.7919	0.9889	0.5996	0.5095
mRNFL-II	0.8440	0.9699	0.7249	0.6801
mRNFL-TI	0.8533	0.8934	0.8513	0.9205
mRNFL-SO	**<0.0001**	**<0.0001**	**0.0111**	0.1454
mRNFL-NO	**0.0301**	**0.0007**	0.6157	**<0.0001**
mRNFL-IO	0.1542	0.0699	0.7576	0.1302
mRNFL-TO	0.6321	0.6268	0.7774	0.8816

mRNFL: macular Retinal Nerve Fiber Layer; VHL: Von Hippel–Lindau; RH: Retinal Hemangioblastoma; mRNFL-Vol: macular Volume of Retinal Nerve Fiber Layer; mRNFL-C: Central sector of macular Retinal Nerve Fiber Layer; mRNFL-SI: Superior-Inner sector of macular Retinal Nerve Fiber Layer; mRNFL-NI: Nasal-Inner sector of macular Retinal Nerve Fiber Layer; RNFL-II: Inferior-Inner sector of macular Retinal Nerve Fiber Layer; mRNFL-TI: Temporal-Inner sector of macular Retinal Nerve Fiber Layer; mRNFL-SO: Superior-Outer sector of macular Retinal Nerve Fiber Layer; mRNFL-NO: Nasal-Outer sector of macular Retinal Nerve Fiber Layer; mRNFL-IO: Inferior-Outer sector of macular Retinal Nerve Fiber Layer; mRNFL-TO: Temporal-Outer sector of macular Retinal Nerve Fiber Layer. *p*-value: statistically significant values (*p* < 0.05) in bold.

**Table 6:** Volume and thickness of GCL in the analyzed groups.

GCL	VHL	VHL without RH	VHL with RH	Controls
GCL-Vol (mm^3^: mean ± SD)	1.111 ± 0.078	1.095 ± 0.078	1.138 ± 0.072	1.135 ± 0.072
GCL-C (μm: mean ± SD)	15.6 ± 3.7	16.1 ± 3.6	14.9 ± 4.0	14.6 ± 3.1
GCL-SI (μm: mean ± SD)	53.7 ± 4.4	53.0 ± 4.8	55.0 ± 3.5	54.5 ± 3.1
GCL-NI (μm: mean ± SD)	52.6 ± 4.9	52.4 ± 5.3	53.0 ± 4.0	52.4 ± 4.2
GCL-II (μm: mean ± SD)	53.3 ± 4.7	52.9 ± 4.8	53.9 ± 4.6	54.3 ± 3.4
GCL-TI (μm: mean ± SD)	49.3 ± 5.0	48.8 ± 5.4	50.4 ± 4.0	50.8 ± 3.3
GCL-SO (μm: mean ± SD)	35.1 ± 2.5	34.6 ± 2.6	36.0 ± 2.3	35.6 ± 2.8
GCL-NO (μm: mean ± SD)	38.9 ± 3.0	38.1 ± 2.9	40.3 ± 2.7	39.4 ± 3.3
GCL-IO (μm: mean ± SD)	34.2 ± 2.7	33.7 ± 2.6	35.0 ± 2.9	34.9 ± 2.7
GCL-TO (μm: mean ± SD)	37.1 ± 3.8	36.5 ± 3.7	38.2 ± 3.8	39.0 ± 3.7

GCL: Ganglion Cell Layer; VHL: Von Hippel–Lindau; RH: Retinal Hemangioblastoma; SD: Standard Deviation; GCL-Vol: Volume of ganglion cell layer; GCL-C: Central sector of ganglion cell layer; GCL-SI: Superior-Inner sector of ganglion cell layer; GCL-NI: Nasal-Inner sector of ganglion cell layer; GCL-II: Inferior-Inner sector of ganglion cell layer; GCL-TI: Temporal-Inner sector of ganglion cell layer; GCL-SO: Superior-Outer sector of ganglion cell layer; GCL-NO: Nasal-Outer sector of ganglion cell layer; GCL-IO: Inferior-Outer sector of ganglion cell layer; GCL-TO: Temporal-Outer sector of ganglion cell layer.

**Table 7:** Statistical significance (*p*-value) of GCL thickness in the analyzed groups.

GCL	VHL vs. Controls	VHL without RH vs. Controls	VHL with RH vs. Controls	VHL without RH vs. VHL with RH
GCL-Vol	0.1533	**0.0412**	0.9601	0.0561
GCL-C	0.2316	0.1190	0.7938	0.2478
GCL-SI	0.3452	0.1092	0.6695	0.0665
GCL-NI	0.7896	0.9930	0.5061	0.5289
GCL-II	0.2684	0.1674	0.7283	0.3602
GCL-TI	0.1035	**0.0381**	0.6938	0.1291
GCL-SO	0.5576	0.2850	0.6962	0.1855
GCL-NO	0.5413	0.1677	0.3898	0.0580
GCL-IO	0.4439	0.2373	0.8842	0.2315
GCL-TO	**0.0212**	**0.0073**	0.3844	0.1068

GCL: Ganglion Cell Layer; VHL: Von Hippel–Lindau; RH: Retinal Hemangioblastoma; GCL-Vol: Volume of ganglion cell layer; GCL-C: Central sector of ganglion cell layer; GCL-SI: Superior-Inner sector of ganglion cell layer; GCL-NI: Nasal-Inner sector of ganglion cell layer; GCL-II: Inferior-Inner sector of ganglion cell layer; GCL-TI: Temporal-Inner sector of ganglion cell layer; GCL-SO: Superior-Outer sector of ganglion cell layer; GCL-NO: Nasal-Outer sector of ganglion cell layer; GCL-IO: Inferior-Outer sector of ganglion cell layer; GCL-TO: Temporal-Outer sector of ganglion cell layer. *p*-value: statistically significant values (*p* < 0.05) in bold.

**Table 8:** BCVA and CRT in the analyzed groups.

BCVA and CRT	VHL	VHL without RH	VHL with RH	Controls
BCVA (letters: mean± SD)	85.5 ± 2.4	85.2 ± 2.3	85.9 ± 2.5	87.6 ± 3.4
CRT (μm: mean ± SD)	273.0 ± 18.2	274.6 ± 16.4	270.2 ± 21.1	272.6 ± 18.4

BCVA: Best Corrected Visual Acuity; CRT: Central Retinal Thickness; VHL: VHL: Von Hippel–Lindau; RH: Retinal Hemangioblastoma.

**Table 9:** Statistical significance (*p*-value) BCVA and CRT in the analyzed groups.

BCVA and CRT	VHL vs. Controls	VHL without RH vs. Controls	VHL with RH vs. Controls	VHL without RH vs. VHL with RH
BCVA	**0.0007**	**0.0010**	**0.0461**	0.2732
CRT	0.9359	0.6553	0.6030	0.3763

BCVA: Best Corrected Visual Acuity; CRT: Central Retinal Thickness; VHL: VHL: Von Hippel–Lindau; RH: Retinal Hemangioblastoma. *p*-value: statistically significant values (*p* < 0.05) in bold.

**Table 10:** HRF in internal, external and total retina in the analyzed groups.

Groups	HRF IR	HRF OR	HRF Total Retina
VHL (mean ± SD)	28.4 ± 8.1	7.5 ± 2.6	36.0 ± 9.3
VHL without RH (mean ± SD)	29.7 ± 7.7	7.8 ± 2.5	37.5 ± 8.8
VHL with RH (mean ± SD)	26.2 ± 8.5	7.0 ± 2.8	33.2 ± 9.8
Controls (mean ± SD)	24.9 ± 7.7	8.0 ± 2.5	32.9 ± 8.9

HRF: Hyperreflective Retinal Foci, IR: Inner Retina, OR: Outer Retina, VHL: Von Hippel–Lindau, RH: Retinal Hemangioblastoma; SD: Standard Deviation.

**Table 11:** Statistical significance (*p*-value) of HRF in internal, external and total retina in the analyzed groups.

Comparison between Groups	HRF IR	HRF OR	HRF Total Retina
VHL vs. Controls	0.0554	0.3985	0.1505
VHL without RH vs. Controls	**0.0121**	0.7616	**0.0302**
VHL with RH vs. Controls	0.5735	0.1798	0.9143
VHL without RH vs. VHL with RH	0.1086	0.2295	0.0677

HRF: Hyperreflective Retinal Foci, IR: Inner Retina, OR: Outer Retina, VHL: Von Hippel–Lindau, RH: Retinal Hemangioblastoma. *p*-value: statistically significant values (*p* < 0.05) in bold.

## Data Availability

The data presented in this study are available in the article. Eventual additional data are available on request from the corresponding author.
